# The association between Google Trends suicide-related Internet searches and self-harm hospitalizations and suicide mortality in Canada

**DOI:** 10.24095/hpcdp.46.1.04

**Published:** 2026-01

**Authors:** Parisa Khodabandehloo, Justin J. Lang, Margot Henry, Gisle Contreras, Wendy Thompson, Li Liu, Raelyne L. Dopko

**Affiliations:** 1 Centre for Surveillance and Applied Research, Public Health Agency of Canada, Ottawa, Ontario, Canada; 2 School of Epidemiology and Public Health, Faculty of Medicine, University of Ottawa, Ottawa, Ontario, Canada; 3 Alliance for Research in Exercise, Nutrition and Activity (ARENA), Allied Health and Human Performance, University of South Australia, Adelaide, South Australia, Australia; 4 Science and Policy Integration Branch, Public Health Agency of Canada, Ottawa, Ontario, Canada

**Keywords:** suicide, intentional self-harm, Canada, surveillance, Google Trends

## Abstract

In this ecological study we examined associations between Google Trends (GT) suicide-related Internet searches and intentional self-harm hospitalizations and suicide mortality in Canada from 31 December 2017 to 31 March 2022. Hospitalizations and mortality data were from the Discharge Abstract Database and Vital Statistics - Death database. Cross-correlations identified lead periods, adjusted for in negative binomial regressions. GT of the search term “how to kill yourself” showed weak positive associations with self-harm hospitalizations. GT of the search terms “commit suicide,” “how to commit suicide” and “how to kill yourself” showed weak positive associations with suicide mortality. Additional research is needed to determine the usefulness of GT in monitoring self-harm and suicide.

**Highlights**

- We explored associations between Google Trends suicide-related searches and intentional self-harm hospitalizations and suicide mortality in Canada.
- Google Trends search terms included “suicide,” “commit suicide,” “how to commit suicide” and “how to kill yourself.”
- The search terms “commit suicide,” “how to commit suicide” and “how to kill yourself” were weakly but positively associated with suicide mortality.
- “How to kill yourself” was weakly but positively associated with selfharm hospitalizations.

## Introduction

Approximately 4500 suicide deaths occur annually in Canada,^1^ and 61 individuals per 100000 are hospitalized due to self-harm,^2^ representing a significant public health issue. Hospital records are typically delayed by 6 to 12 months while mortality records can take up to 2 years because of lengthy coroner investigations.^3^-^5^ Lack of timely data is a barrier to the health surveillance needed to inform rapid resource allocation and public health response.

Google Trends (GT) is a publicly available and deidentified repository of trends and patterns for the search terms used in the Google Search engine (Google, Mountain View, CA, US). These data are available in near real-time (i.e. within the hour), and their use has been proposed as a way to supplement surveillance efforts. Studies published since 2009 have found GT search volumes to be positively associated with infectious disease occurrences like influenza,^6^ HIV,^7^ Lyme disease,^8^ syphilis^9^ and measles.^10^ The potential of GT has also drawn the attention of mental health researchers; a study conducted in the USA that linked suicide mortality to Internet search terms such as “commit suicide,” “how to suicide” and “suicide prevention” suggested that GT could be used to determine heightened suicide risk.^11^ Suicide mortality was found to be positively associated with the GT of the search term “suicide” and negatively with the GT of the search term “depression” in England between 2004 and 2013.^12^

Not all research has demonstrated significant findings.^13^ A Japanese study concluded that the terms “suicide” and “suicide method” were not associated with suicide mortality.^14^ A study conducted in Austria, Germany, Switzerland and the USA reported some temporal associations between GT and suicide rates, but cautioned that the findings were highly variable across and within countries, underscoring the low validity of GT for forecasting national suicide mortality rates.^15^

The objective of our study was to report the associations between suicide-related Internet searches and intentional self-harm hospitalizations and suicide mortality in Canada from December 2017 to March 2022.

## Methods

***Google Trends***

Suicide-related Internet search volume in Canada was gathered using GT, a publicly available online tool that provides deidentified search volumes for a specific topic, at a specified time and location (i.e. provinces and some larger cities).^16^ Instead of showing absolute search numbers, GT calculates a query share for search terms, normalizing the data.^16^ In other words, the number of searches for a term is calculated as a proportion of the total number of searches at a specific time and location. The data are then standardized and scaled by assigning values from 0 to 100,^17^ with higher values indicating greater popularity. A score of 0 reflects low or no search volume.^16^

We investigated which suicide-related terms or phrases had been used in previous studies. After removing terms with missing GT (index score 0),^18^ we decided on four English terms: “suicide,” “commit suicide,” “how to commit suicide” and “how to kill yourself.” Data for these terms from 31 December 2017 to 31 March 2022 were extracted from GT using the *gtrendsR* package.^19^ This period was selected to match available suicide-related outcome data.

***Intentional self-harm hospitalizations***

We used the Canadian Institute for Health Information’s Discharge Abstract Database (DAD) to obtain daily intentional self-harm hospitalizations among individuals aged 10 years or older from all the provinces and territories except Quebec. The DAD records administrative, clinical and demographic hospital release details and classifies diagnostic outcomes, symptoms and procedures during hospitalization^20^ using *International Statistical Classification of Diseases and Related Health Problems, 10th Revision, Canada* (ICD-10-CA) codes. Intentional self-harm events from 31 December 2017 to 31 March 2022 were identified using codes X60–X84 and Y87.0.^21^ These data were aggregated to generate weekly counts.

***Suicide mortality***

We obtained suicide mortality data for individuals aged 10 years and older from the provisional monthly updated Canadian Vital Statistics - Death database (CVSD), which collects demographic and medical cause of death data from every provincial and territorial vital statistics registry (except Yukon).^22^ Weekly suicide counts from 31 December 2017 to 31 March 2022, rounded to base 5 to avoid identifying individuals, were extracted using ICD-10 codes X60–X84 and Y87.0.

***Statistical analysis***

For each query, GT generates a new random sample at unspecified time intervals; we therefore used a bootstrap method to account for the variability in GT query results. We extracted 100 distinct draws by adding random alphanumeric strings to each term (e.g. “suicide 2vpq8aw50e”) to get new results without altering search data.^12^ Using 100 draws per term, we calculated average weekly GT, which allowed us to produce more reliable weekly time series, thereby minimizing the impact of GT’s sampling variability.

Cross-correlation analyses identified significant time-leads that indicate that shifts in GT precede shifts in suicide-related events. We interpreted the Pearson correlation coefficients as weak (*r* ≤ 0.3), moderate (*r* = 0.4 – 0.6) and strong (*r* ≥ 0.7) effect sizes.^23^

Time-leads with the strongest association were incorporated into negative binomial regressions, estimating incidence rate ratios (IRRs). The dependent variables were suicide-related hospitalizations and mortality counts, while the independent variable was GT search volume of each suicide-related term. This quantified the change in the rate of suicide-related events associated with a unit increase in GT search volume. An IRR greater than 1.00 indicates an increased suicide-related events rate, an IRR of less than 1.00 suggests a decrease, while an IRR of 1.00 implies no effect. Statistical significance was determined (α = 0.05).

Analyses were conducted in R version 4.2.2 (R Foundation for Statistical Computing, Vienna, AT) and SAS Enterprise Guide 7.1 (SAS Institute Inc., Cary, NC, US).

## Results

***Intentional self-harm hospitalizations***

Between 31 December 2017 and 31 March 2022, there were 57336 intentional self-harm hospitalizations across Canada (except Quebec). Of these patients, 28.9% were 10 to 19 years old, 30.1% were 20 to 34 years old, 33.6% were 35 to 64 years old and 7.4% were 65 years or older. Approximately 64.8% of those hospitalized for intentional self-harm were female.

Increases in the GT of “how to kill yourself” was weakly and positively correlated with intentional self-harm hospitalizations (*r* = 0.20). The search terms “suicide,” “commit suicide” and “how to commit suicide” were not significantly correlated with the outcomes (Table 1). 

**Table 1:** Cross-correlation model results of the associations between GT suicide-related search term volumes and intentional self-harm hospitalizations and suicide mortality data, Canada, December 2017–March 2022

Event	Search term	Time-lead in months			
		−3	−2	−1	0
Hospitalizations^c^	Suicide	0.10	0.10	−0.01	0.10^e^
	Commit suicide	−0.10	0.07	0.06	0.12^e^
	How to commit suicide	0.00	0.08^e^	0.04	0.05
	How to kill yourself	−0.02	−0.03	−0.08	0.20^e^^*^
Deaths^d^	Suicide	−0.01	0.08	0.05	0.10^e^
	Commit suicide	0.23^e^^*^	0.20^*^	0.19^*^	0.19^*^
	How to commit suicide	0.12	0.19^*^	0.21^e^^*^	0.19^*^
	How to kill yourself	0.13	0.18^e^^*^	0.05	0.08

**Abbreviation: **GT, Google Trends. 

^a ^ Shown as Pearson correlation coefficients, *r*. 

^b^ The search volume of GT data as a proportion of the total number of searches at a specific time and location standardized and scaled from 0 to 100. 

^c^ Self-harm hospitalization data do not include Quebec data. 

^d^ Death data do not include Yukon data. 

^e^ These indicate the maximum lead pattern of the cross-correlation coefficient for that search term, where lead pattern is the time at which GT changes before suicide-related events. 

* Statistically significant at α = 0.05 .

The negative binomial regression revealed that each one-unit increase in GT of “how to kill yourself” was significantly associated with a 0.16% increase in the self-harm hospitalization rate (IRR = 1.0016; 95% CI: 1.0006–1.0027) (Table 2).

**Table 2:** Negative binomial model results of the associations between GT suicide-related search term volumes and intentional self-harm hospitalizations and suicide mortality data, Canada, December 2017–March 2022

Hospitalizations^b^			Deaths^c^		
Search term (time-lead in months)	IRR	95% CI	Search term (time-lead in months)	IRR	95% CI
Suicide (0)	1.0011	0.9996–1.0026	Suicide (0)	1.0020	0.9994–1.0046
Commit suicide (0)	1.0014	0.9999–1.0030	Commit suicide (3)	1.0048^*^	1.0020–1.0075
How to commit suicide (2)	1.0006	0.9996–1.0017	How to commit suicide (1)	1.0029^*^	1.0011–1.0047
How to kill yourself (0)	1.0016^*^	1.0006–1.0027	How to kill yourself (2)	1.0025^*^	1.0007–1.0044

**Abbreviations: ** CI, confidence interval; GT, Google Trends; IRR, incidence rate ratio. 

^a^ The search volume of GT data as a proportion of the total number of searches at a specific time and location standardized and scaled from 0 to 100. 

^b^ Self-harm hospitalization data do not include Quebec data. 

^c^ Death data do not include Yukon data. 

* Statistically significant at α = 0.05. 

***Suicide mortality***

There were 17670 suicide deaths between 31 December 2017 and 31 March 2022. GT search volumes of “commit suicide” at a 3-month lead period (*r* = 0.23), “how to commit suicide” at a 1-month lead period (*r* = 0.21) and “how to kill yourself” at a 2-month lead period (r = 0.18) were weakly and positively correlated with suicide mortality (Table 1). All GT suicide-related searches, with the exception of “suicide,” demonstrated positive associations with suicide mortality (Table 2). A one-unit increase in the GT search volume of “commit suicide” was significantly associated with a 0.48% increase in the suicide mortality rate (IRR = 1.0048; 95% CI: 1.0020–1.0075). We also found significant associations for “how to commit suicide” and “how to kill yourself.”

## Discussion

Our study results indicate that the GT of the search term “how to kill yourself” was positively but weakly associated with same-month intentional self-harm hospitalizations. We also found that GT of the search terms “how to kill yourself,” “commit suicide” and “how to commit suicide” showed weak positive associations with suicide mortality at time-leads of 2, 3 and 1 months, respectively, suggesting that increases in these GT are associated with increases in suicide mortality 1 to 3 months later. Studies in Japan, Taiwan and the USA found positive associations between suicide mortality and the search terms “suicide,” “commit suicide,” “how to suicide,” “suicide prevention” and “depression.”^11^,^14^,^24^ Our correlation coefficients were lower than those in previous studies,^11^,^13^,^14^ suggesting weaker relationships.

Although weak, the consistent strength and the direction of associations highlight nuanced relationships between GT and suicide-related outcomes. Like previous studies,^14^ our research revealed that not all search terms demonstrate equal utility, regardless of the outcome in terms of suicide-related mortality or hospitalizations. Search terms may reflect differences in intent; broad terms such as “suicide” may encompass searches related to prevention or information, whereas explicit phrases such as “how to kill yourself” likely indicate stronger intent towards self-harm or suicidal behaviour.

We found significant lead patterns, where increasing search volumes preceded increases in suicide-related events. Such consistent temporal associations, and the availability of past-hour GT data, may demonstrate GT’s potential as an indicator of suicide-related outcomes.^11^,^24^ Several international studies have suggested that observing changes in GT could help mobilize suicide prevention and mental health services.^3^,^11^,^12^ When paired with responsive policies—public service announcements about suicide prevention resources^11^ or the deployment of rapid response teams, for example—GT data could carry policy implications by helping to prevent suicides.

However, with the weak associations observed and the lack of Canadian research, further work is needed to assess the relevance of GT for informing suicide prevention efforts in Canada,^3^,^11^ especially at a more local level.

***Limitations***

Study limitations include the lack of transparency by Google on how GT are computed (i.e. indexed and normalized). In addition, the age and sex distributions of Google Search users were unavailable, preventing stratified analyses. This absence of sex- or gender-specific data in GT may limit the applicability of our findings, given that in Canada men are at higher risk of dying by suicide while women are more likely to engage in self-harm.1 Estimates of mortality data may change as coroner investigations are finalized.

We may also have failed to capture nuanced information from Quebec by excluding French terms, limiting generalizability. We also did not have access to Quebec’s hospitalization and Yukon’s mortality data, which may have lowered national estimates for the outcomes and potentially attenuated associations.

Finally, GT records of suicide-related searches may not have reflected the search behaviours of those with suicidal thoughts. Instead, unconnected occurrences, for example, suicides of celebrities or releases of movies such as “Suicide Squad,” may have boosted Internet searches, reducing associations.

Future studies could examine GT in Canadian provinces and larger cities, particularly in Quebec. Future research could also examine GT with suicide clusters and lag times (i.e. where increases in GT follow increases in suicide-related events).

## Conclusion

We investigated associations between four suicide-related Internet search terms and intentional self-harm hospitalizations as well as suicide mortality in Canada. We found weak, but statistically significant associations between the GT of the search term “how to kill yourself” and hospitalizations. Associations between GT and mortality were also weak but slightly stronger and more consistent across terms than the hospitalization findings. These findings provide preliminary support for the utility of GT in suicide surveillance and research.

## Funding

None.

## Conflicts of interest

Justin J. Lang is an Associate Editor-in-Chief and an Associate Scientific Editor of the HPCDP Journal, but recused himself from the review process for this article. The authors have no conflicts of interest to declare.

## Authors’ contributions and statement

PK: Conceptualization, methodology, data curation, formal analysis, investigation, project administration, validation, writing—original draft, writing—review and editing.

JJL: Conceptualization, methodology, project administration, supervision, writing—review and editing.

MH: Formal analysis, methodology, validation, writing—review and editing.

GC: Methodology, writing—review and editing.

WT: Methodology, writing—review and editing.

LL: Methodology, writing—review and editing.

RD: Conceptualization, methodology, project administration, supervision, writing—review and editing.

All authors have read and agreed to the published version of the manuscript.

The content and views expressed in this article are those of the authors and do not necessarily reflect those of the Government of Canada.

## References

[B01] Suicide in Canada [Internet]. Government of Canada.

[B02] Suicide Surveillance Indicator Framework [Internet]. Centre for Surveillance and Applied Research.

[B03] Chai Y, Luo H, Zhang Q, Cheng Q, Yip PS (2019). Developing an early warning system of suicide using Google Trends and media reporting. J Affect Disord.

[B04] Data BC Discharge Abstract Database (hospital separations) data set [Internet]. University of British Columbia.

[B05] Overview of administrative health datasets [Internet]. Alberta Health.

[B06] Ginsberg J, Mohebbi MH, Patel RS, Brammer L, Smolinski MS, Brilliant L (2009). Detecting influenza epidemics using search engine query data. Nature.

[B07] Young SD, Zhang Q (2018). Using search engine big data for predicting new HIV diagnoses. PLoS One.

[B08] ny M, Ferenci T, Sulyok Z, Kegele J, Richter H, lyi-Nagy I, et al (2019). Can Google Trends data improve forecasting of Lyme disease incidence. Zoonoses Public Health.

[B09] Young SD, Torrone EA, Urata J, Aral SO (2018). Using search engine data as a tool to predict syphilis. Epidemiology.

[B10] Samaras L, Sicilia MA, Barriocanal E (2021). Predicting epidemics using search engine data: a comparative study on measles in the largest countries of Europe. BMC Public Health.

[B11] Lester D (2013). Using google searches on the internet to monitor suicidal behavior. J Affect Disord.

[B12] Kristoufek L, Moat HS, Preis T (2016). Estimating suicide occurrence statistics using Google Trends. EPJ Data Sci.

[B13] Page A, Chang SS, Gunnell D (2011). Surveillance of Australian suicidal behaviour using the internet. Aust N Z J Psychiatry.

[B14] Sueki H (2011). Does the volume of Internet searches using suicide-related search terms influence the suicide death rate: data from 2004 to 2009 in Japan. Psychiatry Clin Neurosci.

[B15] Tran US, Andel R, Niederkrotenthaler T, Till B, Ajdacic-Gross V, Voracek M (2017). Low validity of Google Trends for behavioral forecasting of national suicide rates. PLoS One.

[B16] Mountain View (CA): Google; [cited 2023 Aug 24]. Google News Initiative: Basics of Google Trends [Internet].

[B17] Ajbar A, Shepherd TA, Robinson M, Mallen CD, Prior JA (2021). Using Google Trends to assess the impact of Global Public Health Days on online health information-seeking behaviour in Arabian Peninsula. J Egypt Public Health Assoc.

[B18] Mountain View (CA): Google; [cited 2024 Aug 21]. Trends help: FAQ about Google Trends data [Internet].

[B19] Massicotte P, Eddelbuettel D RDocumentation: gtrendsR [Internet]. Massicotte P, Eddelbuettel D.

[B20] Discharge Abstract Database (DAD) metadata [Internet]. Canadian Institute for Health Information.

[B21] (2022). ICD-10-CA reference guide [Internet]. CIHI.

[B22] Add/remove data: provisional weekly death counts, by selected grouped causes of death: Table 13-10-0810-01 [Internet]. Statistics Canada.

[B23] Dancey CP, th ed (2007). Statistics without maths for psychology, 4th ed. Pearson/Prentice Hall.

[B24] Yang AC, Tsai SJ, Huang NE, Peng CK (2011). Association of Internet search trends with suicide death in Taipei City, Taiwan, 2004–2009. jad.

